# Supplementary material for: Trimethylamine N-Oxide Levels Are Associated with Severe Aortic Stenosis and Predict Long-Term Adverse Outcome
Source: J Clin Med. 2023 Jan 4;12(2):407. doi: 10.3390/jcm12020407 (PMC9861904; doi:10.3390/jcm12020407)

Table S1. Clinical characteristics of aortic stenosis patients and control subjects After PSM.

	After PSM		
	AS	Controls	P
	N=126	N=126	
Age, years	73(69-77)	72(68-78)	0.319
BMI, kg/m^2	23.82(20.96-25.39)	23.56(21.33-25.78)	0.823
NYHA Class(%)			**<0.001**
II	17(13.5%)	25(22.7%)	
III	56(44.4%)	8(7.3%)	
IV	53(42.1%)	1(0.9%)	
Male(%)	70(55.6%)	67(53.2%)	0.704
Smoker(%)	24(19.0%)	29(23.0%)	0.440
Hyperlipidemia(%)	16(12.7%)	20(15.9%)	0.471
Diabetes(%)	29(23.0%)	22(17.5%)	0.272
Hypertension(%)	72(57.1%)	73(57.9%)	0.899
Prior stroke(%)	10(7.9%)	7(5.6%)	0.451
PVD(%)	21(16.7%)	18(14.3%)	0.601
COPD(%)	21(16.7%)	14(11.1%)	0.202
Cancer(%)	2(1.6%)	5(4.0%)	0.447
GIbleeding(%)	4(3.2%)	5(4.0%)	1.000
eGFR, ml/ (min*1.73m^2)	78.19(59.01-90.37)	86.48(69.02-97.39)	**0.002**
AST, U/L	24(21-30)	29(24-36)	**<0.001**
EF, %	58.9(46.1-65.1)	64.5(58.4-69.4)	**<0.001**
proBNP, pg/ml	2494(541.575-7703)	316.5 (103.25-5388.75)	**0.003**
cTn, ng/ml	0.028(0.012-0.0475)	0.01(0.007-0.017)	**<0.001**
TMAO, µmol/L	2.97(1.38-6.87)	2.15(1.47-3.31)	**0.018**

Values are n (%), or median (interquartile range). **Bold: statistically significant differences.** PSM = propensity score matching; PVD = peripheral vascular diseases; COPD = chronic obstructive pulmonary disease; GI = gastrointestinal; BMI = body mass index; eGFR = estimated glomerular filtration rate; AST = Aspartate Aminotransferase; EF = Ejection Fraction.

Table S2. Spearman correlation analysis of baseline TMAO and clinical characteristics.

	Aortic Stenosis		No AS	
	Spearman	p	Spearman	p
Age, years	0.270	**<0.001**	0.243	**<0.001**
BMI, kg/m^2	-0.038	0.512	0.066	0.08
NYHA Class	0.028	0.632	0.085	**0.023**
eGFR, ml/ (min*1.73m^2)	-0.332	**<0.001**	-0.246	**<0.001**
Hemoglobin, g/L	-0.224	**<0.001**	-0.002	0.966
ProBNP, pg/ml	0.240	**<0.001**	0.251	**0.007**
cTn, ng/ml	-0.043	0.462	0.029	0.499
EF, %	-0.054	0.352	-0.056	0.136

STS score	0.265	**<0.001**	-	-
Mean gradient, mmHg	0.035	0.544	-	-
AVA, cm2	-0.116	**0.045**	-	-
Max velocity, ms	0.060	0.305	-	-
PASP, mmHg	0.097	0.176	-	-
Calcification Grade	0.030	0.602	-	-

Bold: statistically significant differences.

STS = society of thoracic surgeons; PASP = pulmonary arterial systolic pressure. Abbreviations as in **Figure 1 & 2, Table 1**.

Table S3. Linear regression analysis of baseline TMAO and clinical characteristics.

	Univariate regression		Multivariate linear regression		
	Pearson correlation	p value	Coefficient(95%CI)	beta	p value
Age, years	0.199	**0.003**	0.011(0.001-0.020)	0.127	**0.024**
eGFR, ml/(min*1.73m^2)	-0.387	**<0.001**	-0.007(-0.010--0.005)	-0.322	**<0.001**
Log10(ProBNP, pg/ml)	0.155	**0.015**	-	-	-
Log10(cTn, ng/ml)	0.072	0.157	-	-	-
Hemoglobin, g/L	-0.238	**<0.001**	-	-	-
STS	0.180	**0.006**	-	-	-

Stepwise forward regression analysis was performed to identify predictors of TMAO including variables that p < 0.2 in Univariate Pearson correlation analysis. Bold: statistically significant differences. Common Logarithm of TMAO level is used as a dependent Variable. Abbreviations as in **Figure 2, Table 1 & 3**.

Table S4. Univariate Cox regression model to analyze the correlation between TMAO and other clinical characteristics and All-cause mortality and cardiac mortality.

	All-cause mortality		Cardiac mortality		Non-Cardiac mortality	
	Hazard ratio(95%CI)	P	Hazard ratio(95%CI)	P	Hazard ratio(95%CI)	P
TMAO (continuous)	1.015(1.005-1.026)	**0.002**	1.018(1.006-1.031)	**0.005**	1.012(0.996-1.029)	0.145
TMAO ⩾ 6.91mol/L (Q4 vs Q1-3)	2.065(1.255-3.396)	**0.004**	2.151(1.006-4.596)	**0.048**	2.003(1.036-3.871)	**0.039**
Age >81years (Q4 vs Q1-3)	1.426(0.81-2.51)	0.219	0.755(0.26-2.188)	0.604	2.021(1.019-4.011)	**0.044**
Pure AS	1.572(0.97-2.547)	0.067	1.863(0.887-3.912)	0.100	1.387(0.731-2.631)	0.317
eGFR (continuous)	0.979(0.97-0.989)	**<0.001**	0.968(0.953-0.982)	**<0.001**	0.989(0.976-1.002)	0.106

eGFR<30 ml/min (CKD4-5 vs CKD1-3)	4.728(2.466-9.066)	**<0.001**	6.085(2.456-15.076)	**<0.001**	3.739(1.452-9.627)	**0.006**
STS PROM score (continuous)	1.103(1.073-1.133)	**<0.001**	1.098(1.059-1.139)	**<0.001**	1.108(1.064-1.154)	**<0.001**
STS score>8	2.675(1.645-4.352)	**<0.001**	3.715(1.721-8.017)	**0.001**	2.122(1.12-4.02)	**0.021**
NT-proBNP>2821	2.629(1.544-4.476)	**<0.001**	6.368(2.209-18.36)	**0.001**	1.636(0.856-3.127)	0.136
EF(%)(continuous)	0.981(0.965-0.998)	**0.027**	0.975(0.95-1)	0.052	0.986(0.964-1.008)	0.214
EF<50%	1.793(1.107-2.903)	**0.018**	2.209(1.053-4.636)	**0.036**	1.539(0.813-2.914)	0.186
AVAcm2(continuous)	3.117(1.03-9.434)	**0.044**	4.479(0.902-22.251)	0.067	2.307(0.503-10.589)	0.282
AVAcm2<0.58cm2(median)	0.564(0.344-0.925)	**0.023**	0.602(0.281-1.287)	0.19	0.539(0.281-1.031)	0.062
Mean gradient, mmHg (continuous)	0.977(0.961-0.993)	**0.005**	0.973(0.949-0.998)	**0.036**	0.98(0.96-1)	0.054
Mean gradient>53 mmHg(median)	0.559(0.342-0.916)	**0.021**	0.424(0.192-0.94)	**0.035**	0.674(0.357-1.272)	0.224
Max velocity, m/s(continuous)	0.719(0.525-0.984)	**0.039**	0.7(0.431-1.137)	0.149	0.732(0.485-1.106)	0.139
Max velocity >4.78 m/s(median)	0.557(0.34-0.912)	**0.020**	0.496(0.229-1.077)	0.076	0.604(0.319-1.145)	0.123
Valve Type (Bicuspid vs Tricuspid)	0.491(0.29-0.829)	**0.008**	0.465(0.204-1.056)	0.067	0.509(0.258-1.007)	0.052

Bold: statistically significant differences. Abbreviations as in **Figure 1 & 2, Table 1 & 3**.

Table S5. List of Death for non-cardiac reasons.

No. of Deaths (%)	Cause of death
12(31.6)	Neoplasms
11(28.9)	Respiratory disease
4(10.5)	Renal Diseases
4(10.5)	Neurological Disorders
3(7.9)	Unintentional Injuries
2(5.3)	Bleeding/Thrombosis
1(2.6)	Infection
1(2.6)	Unknown

Figure S1. Flow chart of study population.

AS = aortic stenosis; TMAO = Trimethylamine N-oxide; TAVR = Transcatheter Aortic Valve Replacement.

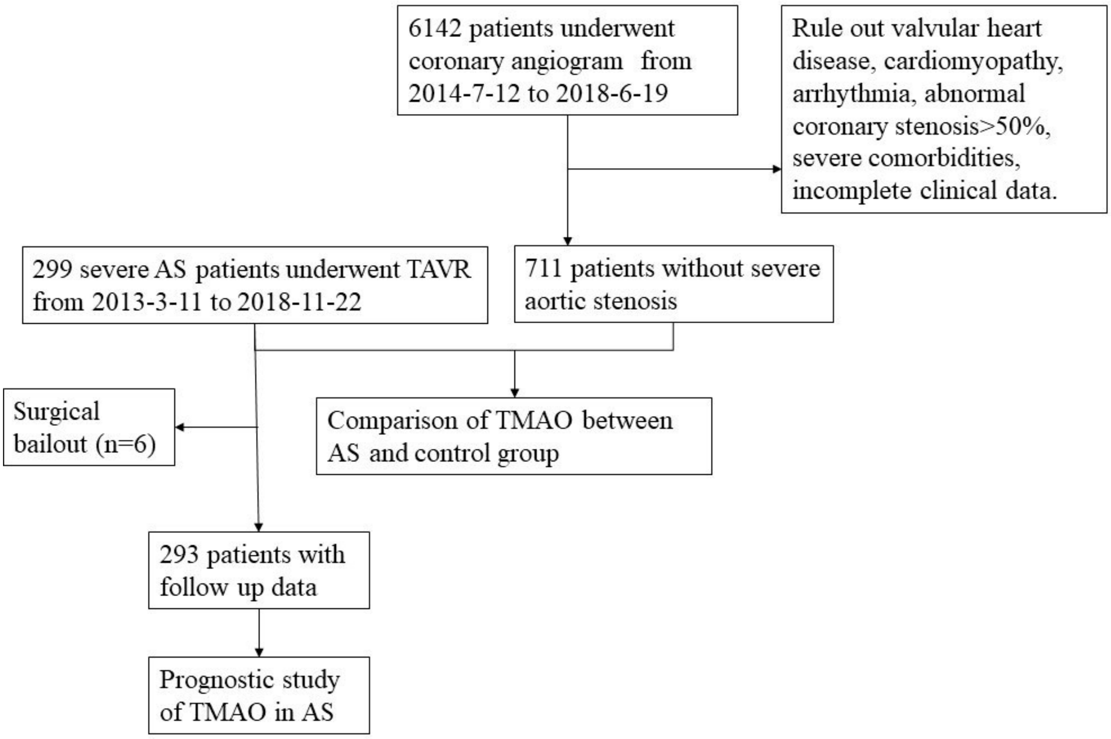

Figure S2. Late Cumulative **(A)** and 2 years **(B)** Event Rate of Mortality According to TMAO quartiles.

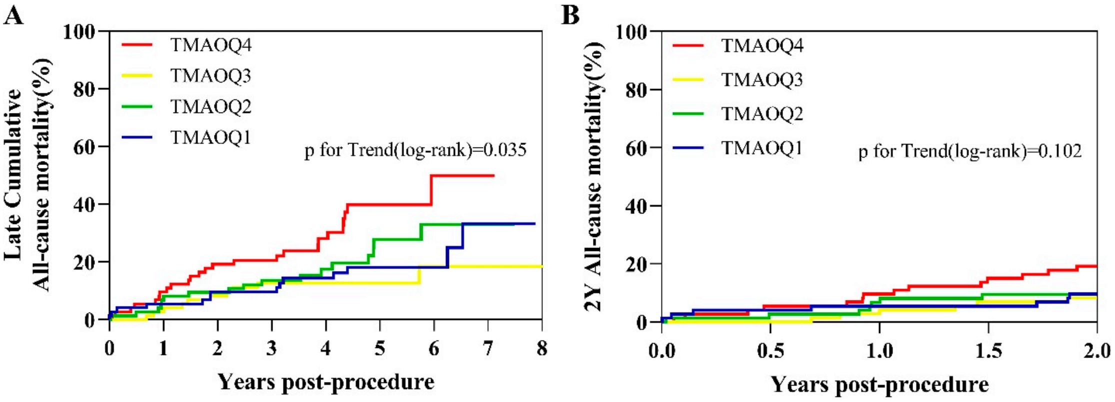

Supplement: Supplementary file 1 [file jcm-12-00407-s001.zip › jcm-2018399-supplementary.pdf]
